# A novel multiplex fluorescent-labeling method for the visualization of mixed-species biofilms *in vitro*

**DOI:** 10.1128/spectrum.00253-24

**Published:** 2024-05-24

**Authors:** Olivia Aherne, Martina Mørch, Roberto Ortiz, Oonagh Shannon, Julia R. Davies

**Affiliations:** 1 Section for Oral Biology and Pathology, Faculty of Odontology and Biofilms Research Center for Biointerfaces, Malmö University, Malmö, Sweden; 2 CR Competence, Lund, Sweden; Innovations Therapeutiques et Resistances, Toulouse, France

**Keywords:** microscopy, staining, live imaging, flow cytometry, confocal spinning disc microscopy, oral bacteria, oral disease, biofilm growth, biofilm detachment, CellTrace

## Abstract

In nature, bacteria usually exist as mixed-species biofilms, where they engage in a range of synergistic and antagonistic interactions that increase their resistance to environmental challenges. Biofilms are a major cause of persistent infections, and dispersal from initial foci can cause new infections at distal sites thus warranting further investigation. Studies of development and spatial interactions in mixed-species biofilms can be challenging due to difficulties in identifying the different bacterial species *in situ*. Here, we apply CellTrace dyes to studies of biofilm bacteria and present a novel application for multiplex labeling, allowing identification of different bacteria in mixed-species, *in vitro* biofilm models. Oral bacteria labeled with CellTrace dyes (far red, yellow, violet, and CFSE [green]) were used to create single- and mixed-species biofilms, which were analyzed with confocal spinning disk microscopy (CSDM). Biofilm supernatants were studied with flow cytometry (FC). Both Gram-positive and Gram-negative bacteria were well labeled and CSDM revealed biofilms with clear morphology and stable staining for up to 4 days. Analysis of CellTrace labeled cells in supernatants using FC showed differences in the biofilm dispersal between bacterial species. Multiplexing with different colored dyes allowed visualization of spatial relationships between bacteria in mixed-species biofilms and relative coverage by the different species was revealed through segmentation of the CSDM images. This novel application, thus, offers a powerful tool for studying structure and composition of mixed-species biofilms *in vitro*.

**IMPORTANCE:**

Although most chronic infections are caused by mixed-species biofilms, much of our knowledge still comes from planktonic cultures of single bacterial species. Studies of formation and development of mixed-species biofilms are, therefore, required. This work describes a method applicable to labeling of bacteria for *in vitro* studies of biofilm structure and dispersal. Critically, labeled bacteria can be multiplexed for identification of different species in mixed-species biofilms using confocal spinning disk microscopy, facilitating investigation of biofilm development and spatial interactions under different environmental conditions. The study is an important step in increasing the tools available for such complex and challenging studies.

## INTRODUCTION

In nature, bacteria are typically found as mixed-species biofilms; complex communities embedded in an extracellular polymeric matrix often attached to a surface (1). The biofilm lifestyle not only affords physical protection but also brings bacteria into close association, promoting synergistic and antagonistic interactions (2–4). Furthermore, biofilm communities can facilitate inter- and intra-species communication through quorum-sensing (5, 6) leading to coordinated gene-expression that increases their resistance to environmental challenges such as low nutrient availability, high temperature, extreme pH, and antimicrobial agents (7, 8). Importantly, microbial biofilms have been proposed to be involved in around 60%–80% of human infections (9). They are also considered a major factor in disease persistence due to the protective properties of the biofilm (10) and the ability of bacteria to detach from initial foci to establish micro-colonies and new infections at distal sites (11).

Although our understanding of biofilm physiology is increasing, current research regarding disease development and progression is still dominated by planktonic culture studies using single bacterial species. This is due both to methodological difficulties in satisfying the nutritional and growth requirements of multiple bacteria and challenges in identifying them in mixed-species biofilms *in vitro*. Lacking the complex bacterial networks and with increased susceptibility to external conditions compared to their biofilm counterparts, planktonic bacteria may not adequately reflect the properties of bacteria in clinical settings (12). Moreover, it is likely that key information regarding biofilm composition, spatial interactions, and the regulation of dispersion will be overlooked. Collectively, this emphasizes the need for more comprehensive and complex, biofilm-based approaches.

Fluorophore-based methods have been widely used as a visualization tool in microbial research. Fluorescent proteins, such as green fluorescent protein (GFP), can be incorporated into the bacterial genome and used for the visualization of biofilm formation and growth (13, 14). Despite the popularity of fluorescent proteins, creation of differently labeled strains for mixed-species applications can be time-consuming and expensive, as well as being ill-suited to anaerobic environments since its fluorescence is dependent on the presence of oxygen (15). Fluorescence *in situ* hybridization (FISH) is another common technique used for visualization of biofilm bacteria (16, 17). However, the requirement for fixation which prevents live-cell imaging and the development of multiple specific probes without cross-reactivity can be difficult due to similarities in 16S-*r*RNA sequences (18). Thus, drawbacks with currently available techniques warrant investigation of new approaches.

Carboxyfluorescein diacetate succinimidyl ester (CFSE) has been widely used in eukaryotic cells as a proliferation marker for flow cytometry (FC) applications (19, 20). The dye is taken up into the cells and conversion by intracellular esterases results in a highly fluorescent, amine-reactive fluorophore that is non-toxic and stable when bound to intracellular proteins. The active fluorophore is maintained in the cytoplasm; therefore, any loss of fluorescent staining should reflect cell division. Although a small number of studies have described its use to enumerate bacteria, monitor their growth rate, and follow their uptake into eukaryotic cells (21–25), CFSE labeling has not been widely exploited in biofilm studies. In addition, a range of differently colored (red, yellow, green, blue, and violet) succinimidyl ester-based dyes (CellTrace, ThermoFisher) is now commercially available, and we, therefore, examined their suitability as a label for bacteria and, in particular, biofilms. Oral bacteria were used to exemplify how CellTrace dyes, in combination with FC and confocal spinning disk microscopy (CSDM), can be used to investigate biofilm formation and dispersal *in vitro*. Most notably, we present a novel application for multiplex labeling to visualize and study mixed-species biofilms.

## RESULTS AND DISCUSSION

### Bacterial labeling with CellTrace dyes

**Fig 1:**
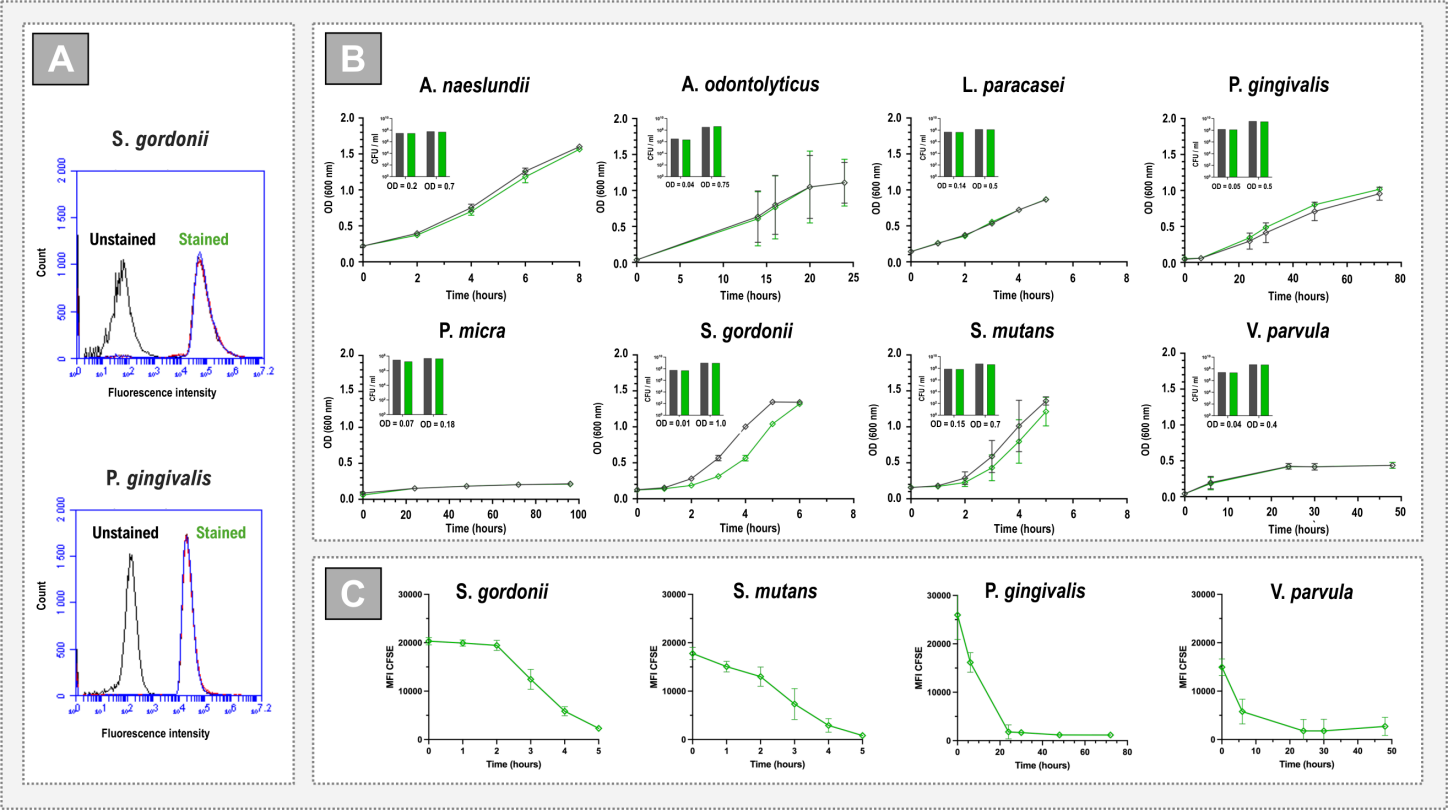
Effects of CellTrace labeling on bacteria. (**A**) FC analysis of bacteria labeled with CellTrace. Gram-positive *S. gordonii* and Gram-negative *P. gingivalis* were labeled for 1 hour with CellTrace™ CFSE (green) and analyzed using FC. Graphs show CFSE staining expressed as the relative fluorescence intensity (*X*-axis) and the relative number of events (counts, *Y*-axis). The data show three experiments carried out using independent biological replicates and a single population of unstained cells was used as a control. (**B**) Growth curve analysis of CellTrace CFSE-labeled planktonic cultures. Cells were pre-labeled for 1 hour, and growth was measured as optical density. Unlabeled cells were used for comparison. Graphs show mean ± SD of three independent biological replicates (except for *P. gingivalis* and *P. micra* where *n* = 2). Bar charts (inserts) show mean ± SD of CFU counts at experiment start and a later stage of growth for each bacterial species. Labeled and unlabeled cells are shown in green and black, respectively. (**C**) Fluorescence intensity of CellTrace™ CFSE-labeled planktonic cultures. Graphs showing mean ± SD of MFI values over time measured using FC. Two representative Gram positive (*S. gordonii* and *S. mutans*) and Gram negative (*P. gingivalis* and *V. parvula*) bacteria are shown.

CellTrace dyes have been widely exploited for the labeling of eukaryotic cells for FC, yet there are few studies describing the use of these reagents for labeling bacteria. Therefore, the capacity of CellTrace CFSE (green) to label Gram-negative and Gram-positive oral bacteria was first examined. Suspensions of *Streptococcus gordonii* and *Porphyromonas gingivalis* were labeled for 1 hour, fixed, and analyzed using FC (Fig. 1A). Histograms of fluorescence intensity revealed high stain coverage in both *S. gordonii* and *P. gingivalis* populations (mean ± SD; 94% ± 1.5% and 98% ± 0.3%, respectively) thus indicating good dye uptake with the chosen staining protocol. Despite a previous report describing poor labeling of *Escherichia coli* with CFSE (attributed to the properties of the Gram-negative cell wall [26]), our data align with studies showing successful CFSE-labeling of non-oral species including *E. coli, Staphylococcus epidermidis,* and *Pseudomonas aeruginosa* (27).

### Effects of CellTrace labeling on growth

Having demonstrated its labeling properties, the effect of CellTrace CFSE on growth of a range of oral bacteria, with different growth rates, was then investigated (Fig. 1B). Assessment of growth, through optical density and colony counts, revealed no major differences between labeled and unlabeled cells for any of the species tested. This confirms that, as seen for eukaryotic cells (19, 20), the presence of the dye did not adversely affect metabolism or cell division processes in the bacteria. In eukaryotic cells, reduction in the fluorescence intensity of CellTrace CFSE has been used to follow cell proliferation (19). Therefore, in parallel to the growth analyses, the fluorescence intensity of four representative bacterial populations was assessed over time using FC (Fig. 1C). Overall, the fluorescence intensity of the population declined in inverse relation to the growth shown in Fig. 1B. For the fast-growing streptococci, fluorescence intensity fell gradually, while the slower growing, *P. gingivalis* and *V. parvula* showed a sharper decrease in MFI over the first 24 hours.

### CDSM visualization of bacterial biofilms labeled with CellTrace

**Fig 2:**
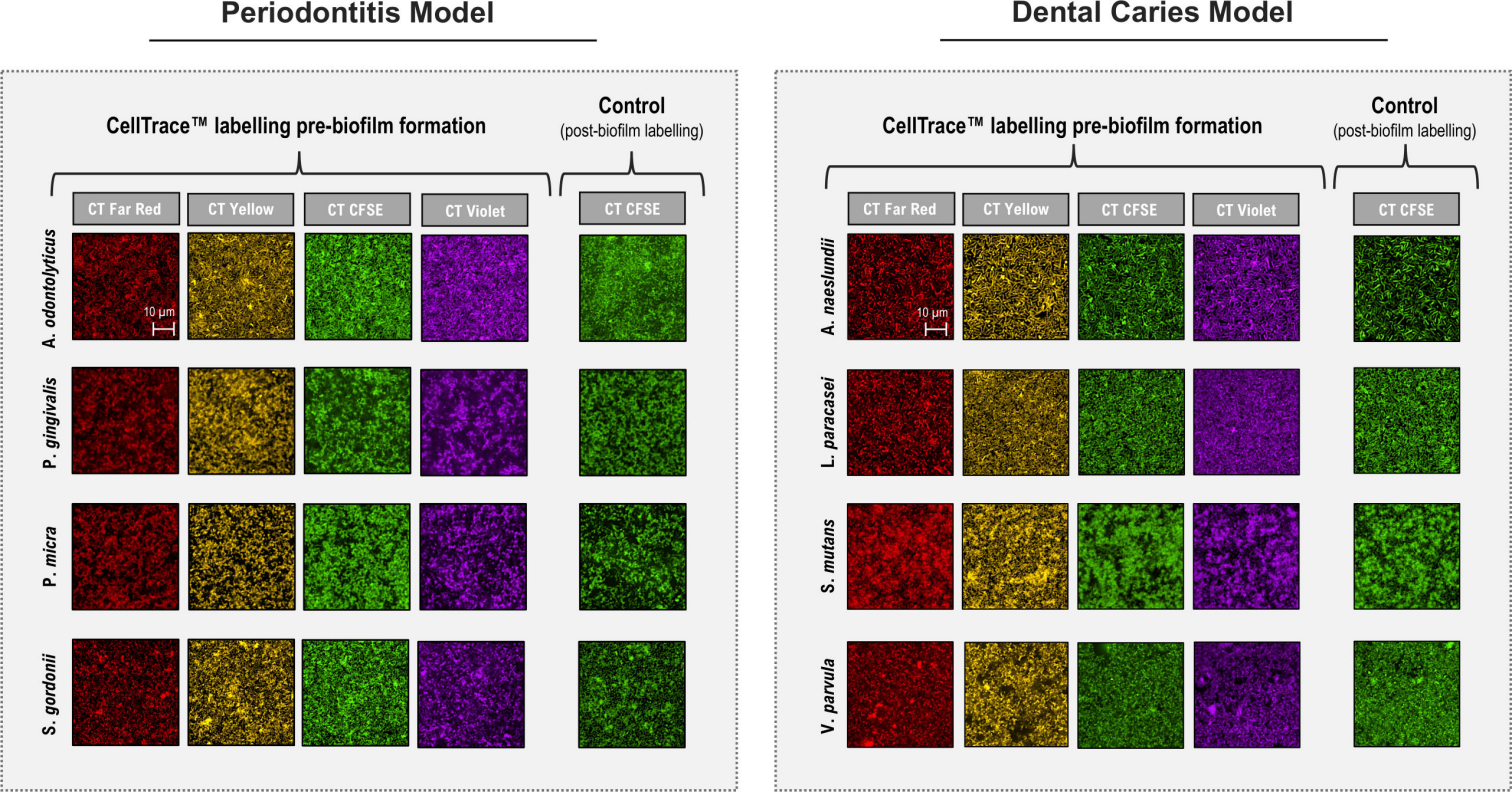
CDSM visualization of SS biofilms labeled with CellTrace. Representative CDSM images of SS biofilms related to oral disease models. Biofilms were formed overnight using unlabeled or CellTrace (far red, yellow, CFSE, or violet)-labeled cells. Unlabeled biofilms were post-stained with CellTrace CFSE for comparison. The periodontitis model is represented by *S. gordonii, A. odontolyticus, P. gingivalis,* and *P. micra,* whereas the dental caries model is comprised of *S. mutans*, *V. parvula, L. paracasei,* and *A. naeslundii*. The scale bar (10 µm) applies to all panels.

No previous studies were identified where CellTrace dyes have been applied to staining and imaging of bacterial biofilms. Therefore, a range of bacteria associated with two oral disease conditions; periodontitis (*S. gordonii, Actinomyces odontolyticus, P. gingivalis,* and *Parvimonas micra*) (28) and caries (*Streptococcus mutans*, *Veillonella parvula, Lacticaseibacillus paracasei,* and *Actinomyces naeslundii*) (29) were individually labeled with four CellTrace colors (red, yellow, green, and violet). Formation of single-species (SS) biofilms was then carried out overnight under anaerobic growth conditions in a protein-rich medium (PRNM) which supports the growth of a wide range of oral bacterial species (30). To ensure that biofilm formation was not affected by the staining, overnight biofilms were created in parallel, using non-stained cells and then post-stained with CellTrace CFSE. The early biofilms were then examined using CSDM to generate single-plane images (Fig. 2). Despite varying levels of bacterial surface coverage between species, CellTrace labeling gave similar results for all colors. High signal-to-noise ratios and well-defined morphologies confirmed the short-term stability and adaptability of the dyes for bacterial labeling. No significant differences were seen between surface coverage of biofilms formed with pre-stained cells compared to post-stained cells for most of the bacteria tested (Table 1). The exception was *V. parvula*. Here, pre-stained cells appeared to give slightly thicker biofilms than the unstained ones, suggesting that, although there was a small difference, CellTrace labeling does not, at least, affect biofilm formation negatively. Thus, we conclude that pre-labeling of the cells, before surface contact, does not appear to have major effects on biofilm formation and provides an advantage over staining biofilms *in situ* with dyes such as Syto 9, as background staining is minimized. We also propose that CellTrace dyes offer a valuable alternative to GFP reporters as visualization tools under anaerobic growth conditions due to their requirement for oxygen for fluorophore maturation (15).

**TABLE 1:** Comparison of surface coverage of SS biofilms labeled with CellTrace CFSE pre- or post-biofilm development^*a*^

	Pre-stained		Post-stained		Significance
	Mean (%)	SD (%)	Mean (%)	SD (%)	
Periodontitis model species					
*A. odontolyticus*	37.84	4.89	45.59	13.40	ns
*P. gingivalis*	37.98	3.64	37.21	5.65	ns
*P. micra*	37.27	6.46	34.96	11.32	ns
*S. gordonii*	41.20	7.90	39.69	6.44	ns
Dental caries model species					
*A. naeslundii*	44.30	5.88	40.27	4.60	ns
*L. paracasei*	34.54	7.34	42.7	7.89	ns
*S. mutans*	41.05	14.71	37.07	7.74	ns
*V. parvula*	41.58	8.22	29.90	12.35	0.0315

^*a*^ Mean % surface coverage of the SS biofilms using cells stained prior to, or after biofilm formation, was compared using a Mann Whitney *U*-test (ns, non-significant difference).

### Bacterial detachment studies using CellTrace-labeled SS biofilms

**Fig 3:**
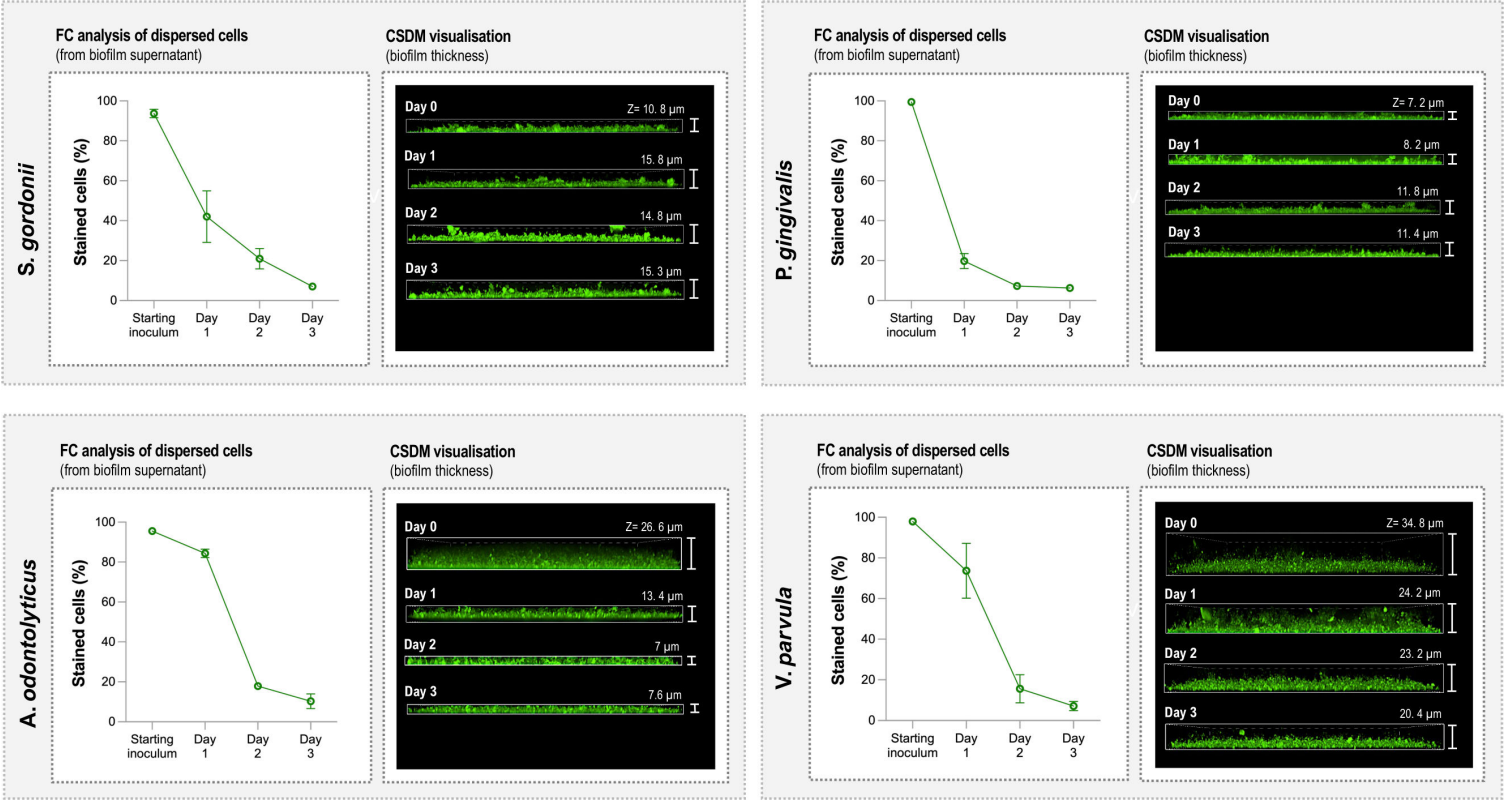
Examination of biofilm structure and cell detachment from SS biofilms over time. Gram-positive (*S. gordonii* and *A. odontolyticus*) and Gram-negative (*P. gingivalis* and *V. parvula*) bacteria labeled with CellTrace CFSE (green) were used to create SS biofilms. Graphs (left panels) show percentages of CFSE-stained cells present in the starting inocula and biofilm supernatants. Supernatants were extracted and analyzed with FC on days 1, 2, and 3. The right panels show representative Z-plane CDSM images of SS biofilms after 4 hours (day 0) and 1, 2, and 3 days.

Having demonstrated that CellTrace dyes can be used to visualize biofilms with CSDM and identify bacteria using FC, we then applied CellTrace-labeling to studying bacterial detachment from biofilms over a 3-day period. Using SS biofilms of Gram-positive (*S. gordonii* and *A. odontolyticus*) or Gram-negative (*P. gingivalis* and *V. parvula*) species, dispersal of cells into the supernatant was followed with FC, and representative z-stack CSDM images were used to study morphology of the corresponding biofilms (Fig. 3). Overall, all species formed stable biofilm structures and showed a steady decrease in the percentage of stained cells present in the supernatant over time. Despite both being regarded as primary colonizers of the tooth surface (31), biofilm structure varied between the two Gram-positive species on day 1, with *S. gordonii* forming relatively thin, dense biofilms while those of *A. odontolyticus* appeared as much thicker and looser structures. Over the following 3 days, the structure of the stained *S. gordonii* biofilms remained relatively stable whereas those of *A. odontolyticus* became initially thinner and more compact, before stabilizing. Larger inter-species variation was seen in the supernatants, with the percentage of detached cells labeled with CellTrace for *S. gordonii* falling to 42% ± 13% on day 1 compared to 84% ± 2% for A. *odontolyticus*. A similar picture was seen for the Gram-negative bacteria, with the *P. gingivalis* and *V. parvula* biofilms resembling those of *S. gordonii* and A. *odontolyticus,* respectively. Similarly, there was a large difference between species in the percentage of stained cells in the supernatant, with 20% ± 4% for *P. gingivalis* and 74% ± 13% for *V. parvula* by day 1 (see Fig. S2 for representative histogram plots).

Collectively, these data highlight how dyes can be used to study temporal changes biofilm in structure and integrity. In particular, we showed that changes identified in biofilm thickness using CSDM can be correlated with the release of labeled cells into the biofilm supernatant measured with FC. As such, this approach would be particularly valuable in investigations of the effects of substances (e.g., quorum-sensing molecules or host proteins) on biofilm dispersal (10) as well as in the identification of agents for biofilm eradication. Similarly, examination of supernatants through FC can shed light on the rate of cell division and dispersal rates within the biofilm. In eukaryotic cells, FC can be used to detect CellTrace for up to eight division cycles as intracellular levels of CellTrace are halved at each cell division (32, 33). In this study, changes in the levels of the dye in individual bacteria could not easily be discerned. Yet as >95% of the bacteria in the starting inoculum were labeled, appearance rate of unstained cells in the supernatant over the 3-day observation period, combined with the relatively constant biofilm thickness, should reflect cell division and dispersal within the biofilm. Thus, in this study, *S. gordonii* appeared to have faster rates of biofilm growth and division than *A. odontolyticus*, *P. gingivalis,* and *V. parvula*, as demonstrated by a more rapid increase in the proportion of unstained cells appearing in the supernatant. An alternative explanation for the appearance of unstained cells in the supernatant could be the loss of dye due to metabolic processes occurring within the dispersed bacterial cells. However, the fact that the bacteria remaining in the biofilms are still stained after 3 days suggests that it is well retained in viable cells over time. Although further experiments will be required to fully clarify these issues, the data presented highlight how CellTrace dyes, in combination with CSDM and FC, could be used to provide insight into the effects of, for instance, nutritional conditions on biofilm growth of different bacterial species.

### Assessment of CellTrace staining as a marker of bacterial viability

**Fig 4:**
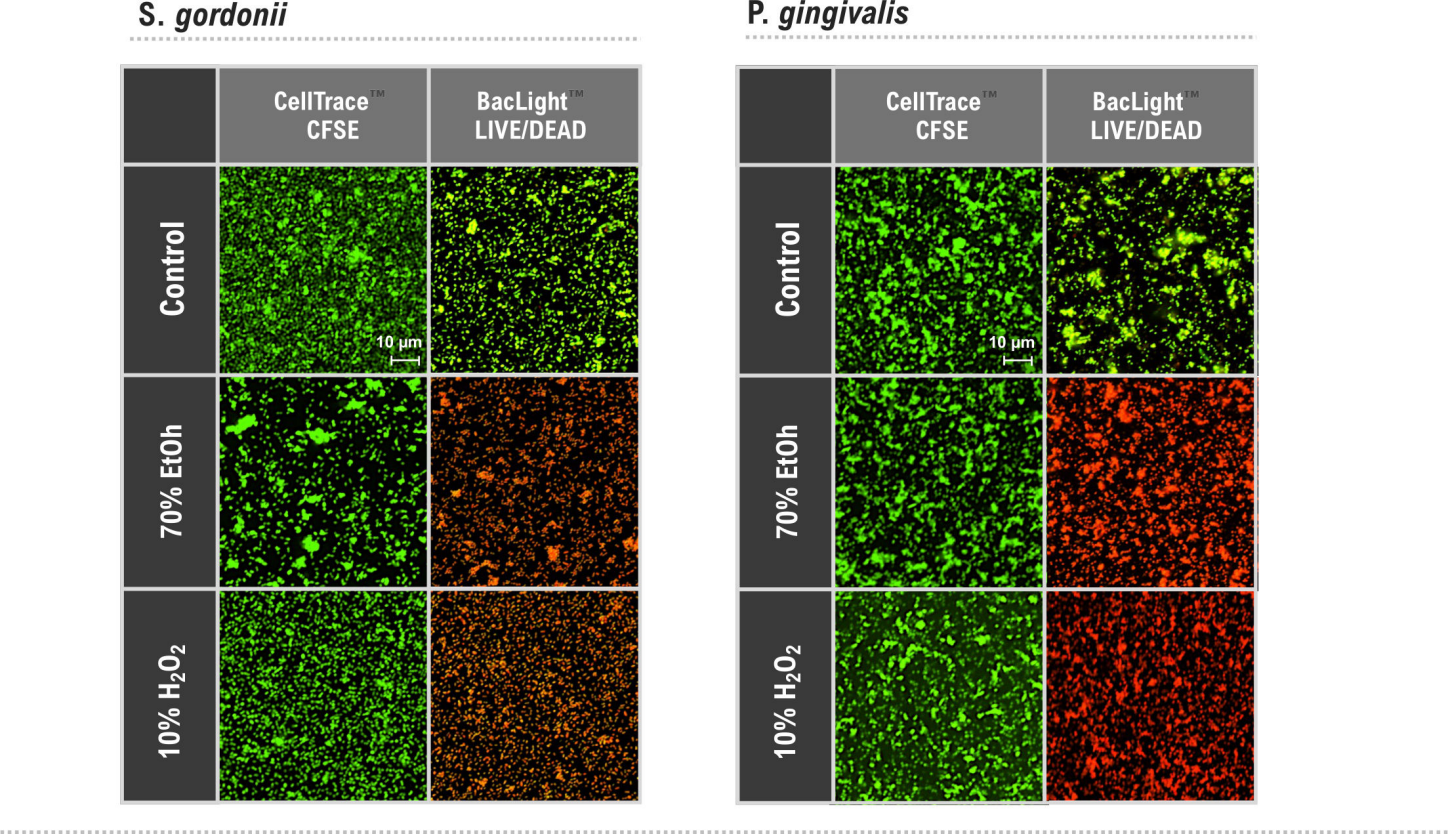
Assessment of CellTrace staining as a marker of bacterial viability in biofilms representative CDSM and CLSM images of overnight SS biofilms stained with CellTrace CFSE (green) or BacLight LIVE/DEAD stain. Biofilms of *S. gordonii* and *P. gingivalis* were maintained in PRNM (control) or treated with 70% ethanol or H_2_O_2_ for 1 hour. The scale bar (10 µm) applies to all panels.

Since in eukaryotic cells, the conversion of CFSE to its fluorescent derivative requires the presence of active intracellular enzymes, we investigated how CellTrace staining was related to bacterial viability. In a previous study, no correlation was seen between the number of active cells and level of CFSE staining in planktonic populations of *Aeromonas hydrophilia*, *Bacillus subtilis*, *E. coli*, *P. aeruginosa,* or *S. epidermidis* (27). Therefore, to determine whether CellTrace staining reflected the viability of sessile oral bacteria, SS biofilms of *S. gordonii* and *P. gingivalis* were treated with 70% ethanol or 10% H_2_O_2_ and subsequently exposed to CellTrace CFSE or BacLight LIVE/DEAD stain (Fig. 4). Despite red staining with the LIVE/DEAD stain indicating damaged or dead cells, treated biofilms stained heavily with CellTrace CFSE indicating that the dye is not a reliable marker for viability in biofilms. This confirms the results obtained for planktonic cells where mechanisms proposed for the effect included non-enzymatic reaction of the stain with intracellular amine groups or binding of the stain to free amine groups on the cell surface (27).

### Discrimination between bacteria in mixed-species biofilms using multiplex CellTrace labeling

**Fig 5:**
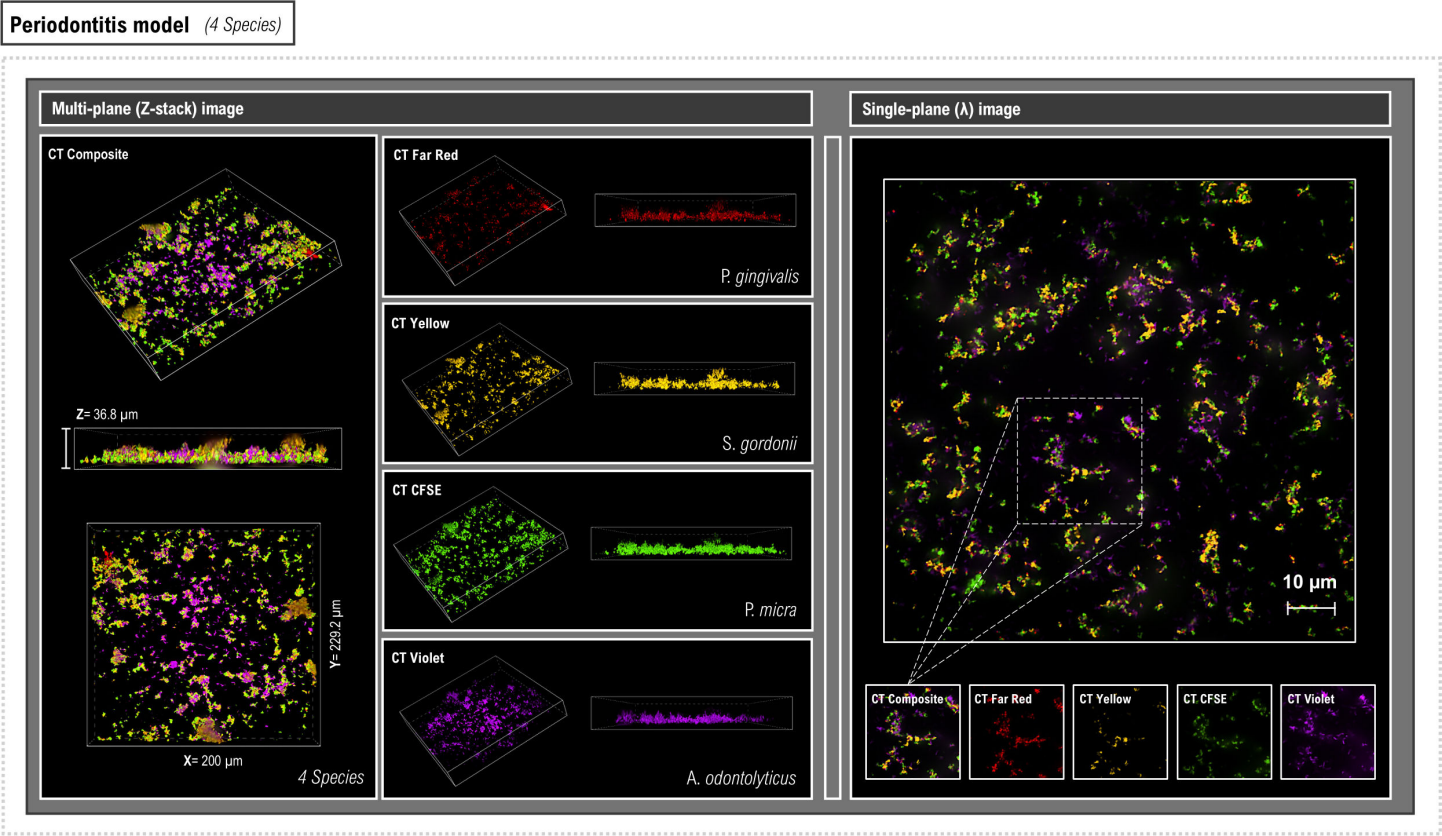
Visualization of MS biofilms representing a periodontitis model labeled by multiplexing CellTrace dyes. Representative CDSM Z-stack (left) and single-plane (right) images of overnight MS biofilms stained with CellTrace dyes. A unique CellTrace color was used to label four species (*P. gingivalis* [red], *S. gordonii* [yellow], *P. micra* [green], and *A. odontolyticus* [violet]). Both multiplex (4-color compilation) and the color-segmented images are shown. The percentage coverage for each species within the biofilm models was calculated using a region of interest (ROI) within the delineated area in the single-plane image. Z-stack images were used to provide information regarding bacterial spatial location and relationships within the overall biofilm.

**Fig 6:**
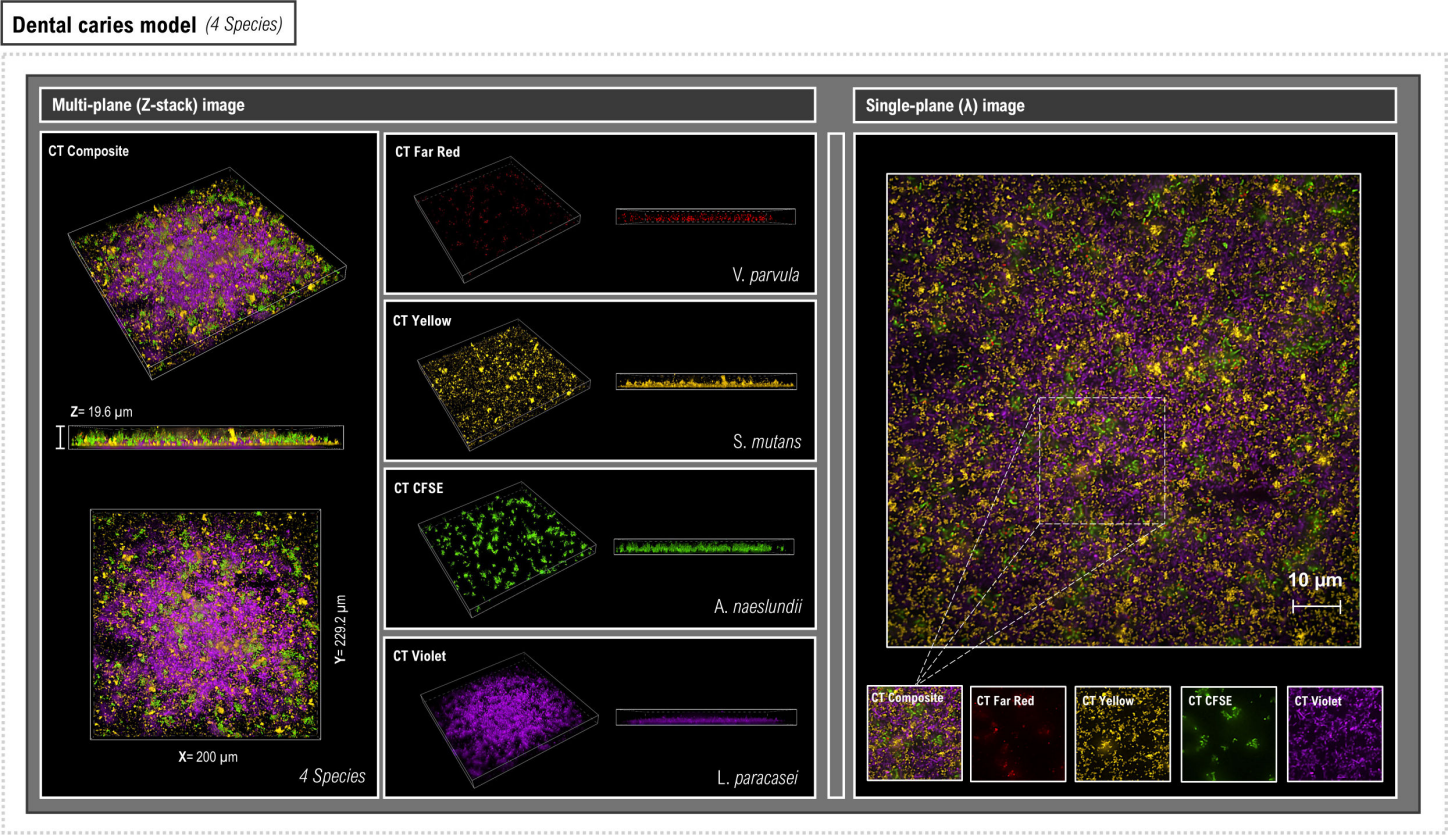
Visualization of MS biofilms representing a dental caries model labeled by multiplexing CellTrace dyes. Representative CDSM Z-stack (left) and single-plane (right) images of overnight MS biofilms stained with CellTrace dyes. A unique CellTrace color was used to label four species (*V. parvula* [red], *S. mutans* [yellow], *A. naeslundii* [green], and *L. paracasei* [violet]). Both multiplex (4-color compilation) and the color segmented images are shown. The percentage coverage for each species within the biofilm models was calculated using a region of interest (ROI) within the delineated area in the single-plane image. Z-stack images were used to provide information regarding bacterial spatial location and relationships within the overall biofilm.

Since the oral bacteria could be successfully labeled with all the colored CellTrace dyes, we then investigated how these could be multiplexed to allow parallel visualization of different bacteria in *in vitro* mixed-species (MS) biofilms. Each species was labeled with a CellTrace dye (CFSE, far red, yellow, or violet), and they were then used to create oral disease models reflecting either periodontitis (*A. odontolyticus*, *P. gingivalis*, *P. micra,* and *S. gordonii*) (Fig. 5) or dental caries (*A. naeslundii*, *L. paracasei*, *S. mutans,* and *V. parvula*) (Fig. 6). Following overnight incubation, biofilms were visualized for initial analysis of spatial relationships between different species using CSDM single- and Z-plane images. In the periodontitis model, the biofilms were relatively thin and *S. gordonii* and *A. odontolyticus* appeared to be mainly associated with the surface layers. In the caries-associated biofilm, *L. paracasei* and *S. mutans* were mainly found close to the flow-cell surface, whereas another species of *Actinomyces*, *A. naeslundii,* was present in the upper biofilm layers.

Labeling and visualization of the different bacteria then allowed determination of their relative contributions to the MS biofilm. Estimation of the relative coverage of each species was performed on individual channels corresponding to each CellTrace color in the composite image for three independent biological replicates. Within a selected region of interest (ROI), it was shown that all species became incorporated into the periodontitis biofilm in similar amounts giving an overall mean composition of *P. gingivalis* (29% ± 3%), *A. odontolyticus* (29% ± 5.5%), *P. micra* (25% ± 3%), and *S. gordonii* (17% ± 1.5%). In contrast, the caries-associated biofilms were composed mainly of *S. mutans* (41% ± 2%) and *L. paracasei* (47% ± 4%) with low levels of *A. naeslundii* (8% ± 4%) and *V. parvula* (4% ± 1.5%). Thus, we demonstrated that this method is well suited to investigations focusing on the relative contribution of different bacterial species to biofilm models. Additional information regarding the spatial and temporal arrangement of species within the biofilms could be obtained if CellTrace staining was combined with more powerful quantitative image analysis tools such as BiofilmQ (34) or Comstat2 (35).

**Fig 7:**
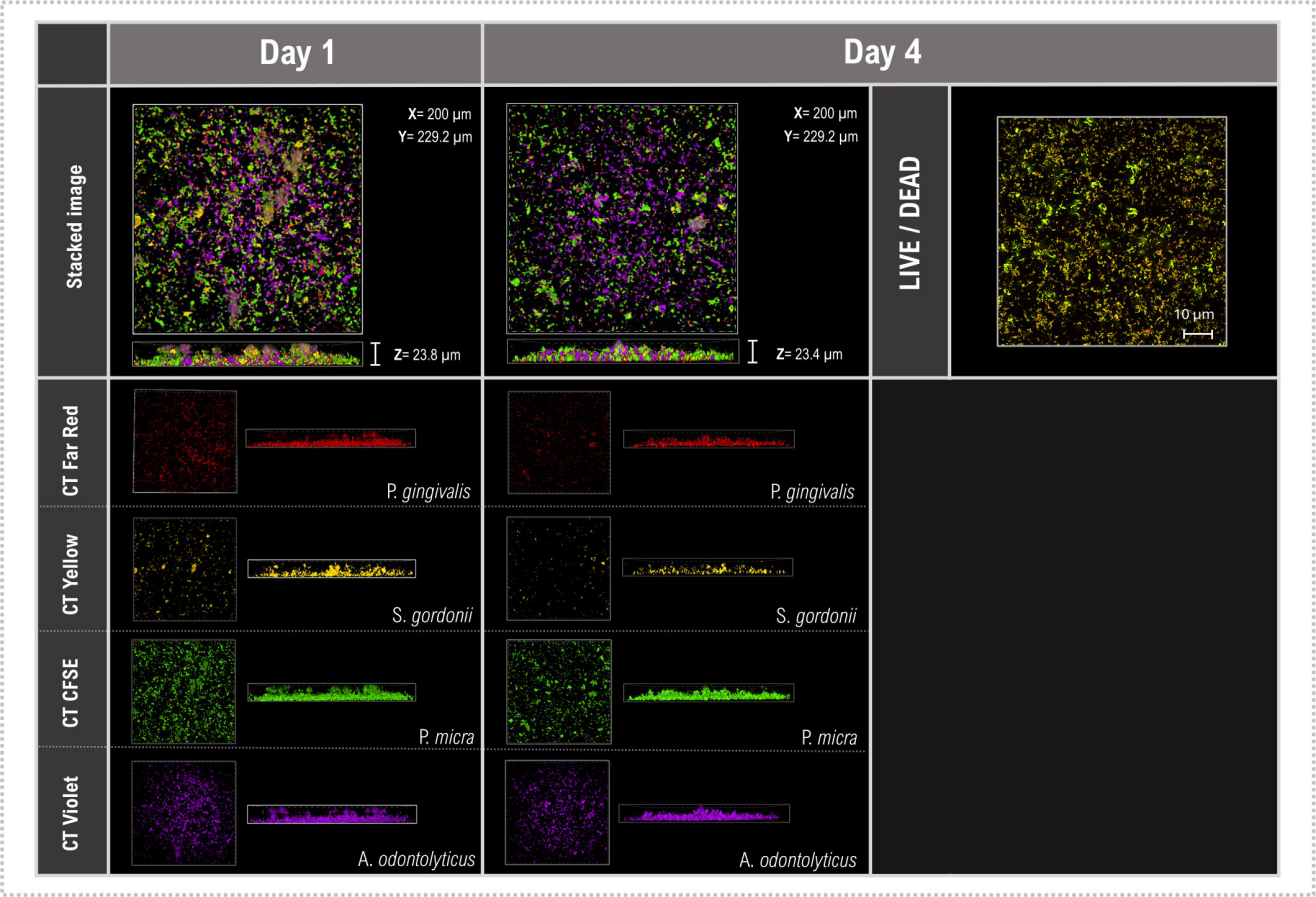
Assessment of CellTrace durability in MS biofilms. Representative CSDM and CLSM images of MS biofilms. Biofilms were created using the periodontitis model (*P. gingivalis* [red], *S. gordonii* [yellow], *P. micra* [green], and *A. odontolyticus* [violet]) and visualized following overnight growth (day 1) and after 4 days through CDSM Z-stack images. Parallel, non-labeled biofilms were also grown, stained with BacLight LIVE/DEAD stain on day 4 and images using CLSM to assess viability. The scale bar (10 µm) applies to all panels.

For future application of this methodology, it is essential that CellTrace dyes are durable within biofilm populations over time, especially in studies examining temporal changes in biofilm composition. Mixed-species biofilms corresponding to the periodontitis model were, therefore, grown and imaged using identical parameters on days 1 and 4 (Fig. 7). No detectable differences in staining intensity were observed between the imaging time points, suggesting that CellTrace dyes remain in the biofilm for at least 4 days. However, over time a reduction in fluorescence intensity due to, for instance, loss of CellTrace dye from actively dividing cells, metabolism of labeled molecules or changes in intracellular pH, could lead to their under-representation in biofilm images, necessitating a complementary method such as brightfield microscopy for longer experiments. Parallel biofilms were stained with BacLight LIVE/DEAD stain after 4 days revealing the majority of the cells to be viable. This result suggests that, as for planktonic cells (36), the dyes can be used for live imaging of biofilm cells. Collectively, these data demonstrate the potential of multiplexing CellTrace dyes to study co-aggregation and spatial relationships between different bacteria during biofilm formation (37) as an alternative to FISH methodology.

### Application of CellTrace dyes to investigation of the effects of local environment on early biofilm formation

**Fig 8:**
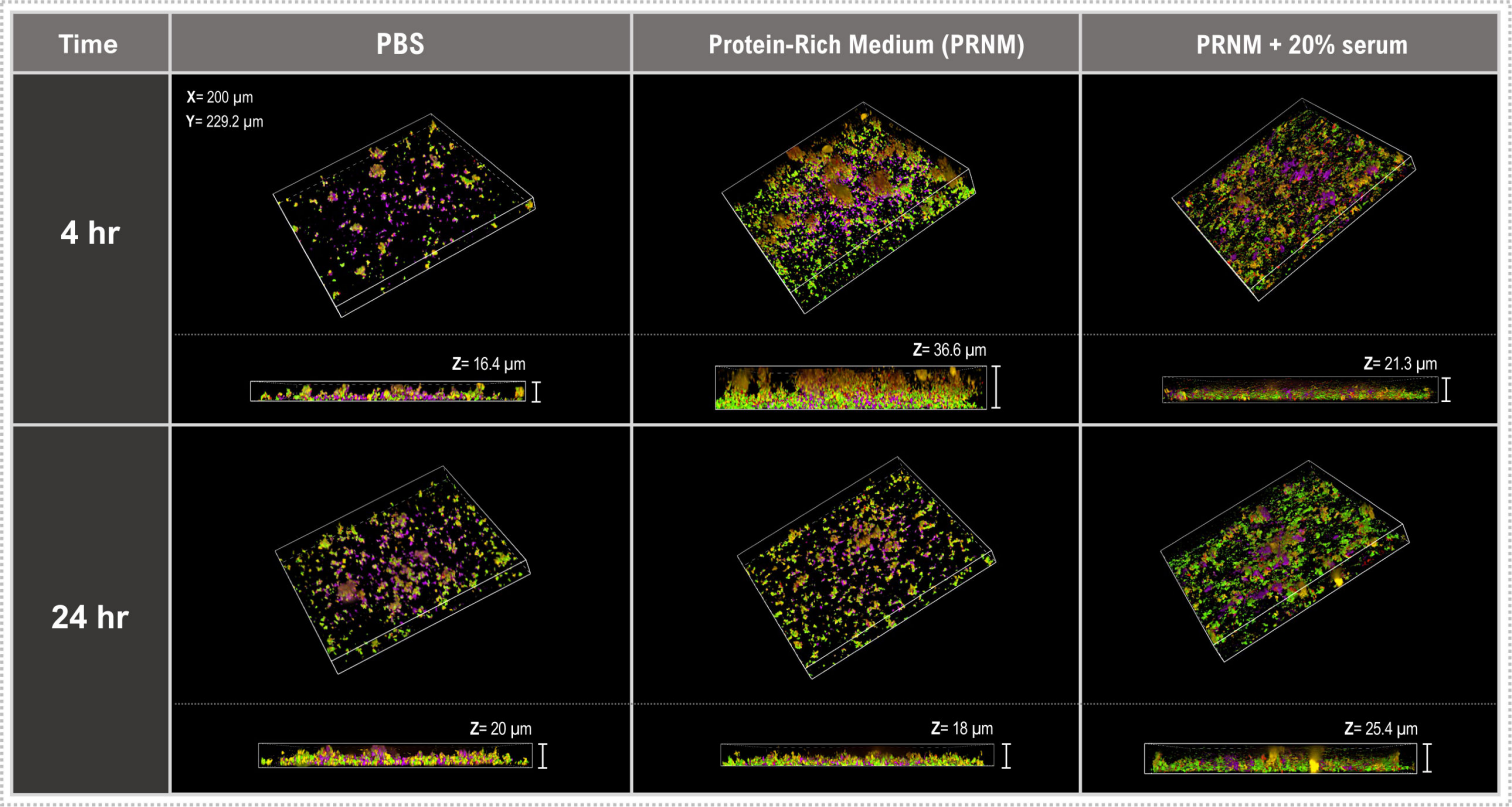
Investigation of the influence of environment on early biofilm formation. Representative CDSM Z-stack images of overnight MS biofilms. Biofilms were created using the periodontitis model (*P. gingivalis* [red], *S. gordonii* [yellow], *P. micra* [green], and *A. odontolyticus* [violet]). They were then grown in either PBS, PRNM, or PRNM + serum, and imaged after 4 or 24 hours.

Differences in the capacity of bacteria to adhere to a surface as well as nutritional conditions are known to play an important role in the determination of the overall biofilm composition and properties over time (38). Therefore, using the multiplexing technique, the influence of time and environmental conditions on early biofilm formation was examined in the periodontitis model (Fig. 8). After centrifugation followed by a 4-hour incubation, bacterial adherence to the flow-cell surface in the presence of PBS buffer was sparse, with isolated compact, aggregates of multiple bacterial species. However, a substantial increase in surface coverage was seen for biofilms developed in PRNM or PRNM + serum. The biofilms also varied in the Z-plane, with PRNM giving rise to thicker more diffuse biofilms than those formed in PBS or PRNM + serum; although after 24 hours, the morphology and thickness of the labeled biofilm were more similar irrespective of the environment. These data agree with previous studies showing that early biofilm formation is highly related to nutritional conditions (39). Overall, this study clearly demonstrates that the CellTrace dyes can be multiplexed to investigate the influence of environmental factors on biofilm formation and the approach could easily be adapted to study, for instance, the role of different proteins in the surface conditioning film on early biofilm formation.

### Conclusion

In conclusion, we have shown that CellTrace dyes represent a simple method of labeling of both Gram-positive and Gram-negative bacteria for biofilm studies. Use of the dyes, in combination with FC and CDSM, opens up possibilities for a variety of studies, including those investigating structural properties of *in vitro* biofilms, as well as mechanisms related to biofilm dispersal. Although CellTrace dyes appear to be non-toxic, staining of dead cells indicates that they are not suitable as a marker of bacterial cell viability. The dyes appear stable within bacterial cells over 4 days, but the results of long-term studies should be interpreted with caution since cellular activities, such as cell division, metabolic activity, or changes in intracellular pH, may reduce fluorescence intensity making quantification of such cells difficult in biofilm images stained with CellTrace alone.

The foremost aspect of our study was the novel application, where we show how CellTrace dyes can be multiplexed to visualize spatial relationships between different bacteria in MS biofilms. Through segmentation of the images, we were also able to study the relative coverage by the different species within the biofilms. Thus, we conclude that this methodology offers enormous potential for furthering our understanding of biofilm development and physiology.

## MATERIALS AND METHODS

### Oral bacterial strains and culture environments

Bacteria used in these studies were *Actinomyces naeslundii* (110BT), *Actinomyces odontolyticus* (G3H), *Fusobacterium nucleatum* (BK:0), *Lacticaseibacillus paracasei* (LC1), *Streptococcus mutans* (B4B), *Streptococcus gordonii* (CW), *Parvimonas micra* (EME), *Porphyromonas gingivalis* (W50d), and *Veillonella parvula* (10BB). All were archived clinical strains originally isolated from the oral cavity. Identification was based on colony morphology on blood agar, as well as phenotypic testing, and was confirmed using MALDI-ToF mass spectroscopy. All strains were stored in skimmed milk (Oxoid Ltd, Hampshire, UK) at −80°C before being taken up on either blood or brucella agar at 37°C under anaerobic conditions (10% H_2_, 5% CO_2_ in N_2_) and grown for up to 7 days.

### CellTrace labeling of bacteria and formation of biofilms

All bacterial suspensions used in this study were prepared by harvesting colonies from freshly prepared blood agar plates and vortexing thoroughly. For planktonic experiments, suspensions (1 × 1 µL loop suspended in 1 mL PBS) were incubated with 2 µL/mL of CellTrace stain (CFSE [carboxyfluorescein-diacetate-succinimidyl ester] green), ThermoFisher Scientific, Sweden) under anaerobic conditions for 1 hour. Following incubation, cells were centrifuged (5,000 rpm, 5 min) and resuspended in PBS to remove any excess dye.

For SS models, bacterial suspensions (3 × 1 µL loops in 1 mL PBS) were labeled with CellTrace stain (CFSE, far red, yellow, or violet) as described above. They were then resuspended in a modified protein-rich nutrient medium (PRNM;, excluding the porcine gastric mucin) developed by Naginyte et al. (30) to support the growth of multiple bacteria in mixed-species consortia. Aliquots (200 µL) of each suspension were then added to channels in an Ibidi IbiTreat VI slide (Ibidi GmbH, Germany) and incubated overnight in a humid chamber at 37°C under anaerobic conditions to initiate biofilm formation. Pre-formed, overnight biofilm controls were washed with PBS and post-stained *in situ* with CellTrace CFSE for 1 hour.

For the MS models, the four component bacteria were labeled individually with a different CellTrace color as described for the SS biofilms (periodontitis: *P. gingivalis* [far red], *S. gordonii* [yellow], *P. micra* [CFSE-green], and *A. odontolyticus* [violet] and dental caries *V. parvula* [far red], *S. mutans* [yellow], *A. naeslundii* [CFSE-green], and *L. paracasei* [violet]). Equal volumes of the bacterial suspensions were then mixed before adding to the Ibidi slides. All experiments were carried out in triplicate using independent biological replicates.

### Growth analysis

For preparation of growth curves, unstained bacteria as well as bacteria labeled for 1 hour with CellTrace CFSE were resuspended in optimal growth medium for each species (*S. gordonii* and *P. gingivalis—*brain heart infusion [BHI]; *A. naeslundii*, *A. odontolyticus*, *L. casei*, *S. mutans—*Todd Hewitt + yeast extract [THYE]; and *V. parvula* and *P. micra—*THYE + 2% yeast extract) and maintained under anaerobic conditions as described above. Aliquots were removed at intervals, and the optical density (OD_600_) was measured. For CFU measurements, serial dilutions were made on blood agar using aliquots taken at the experiment start and a later growth stage (shown in Fig. 1B). Plates were incubated under anaerobic conditions for 4–7 days before counting CFU.

### Flow cytometry analysis

Labeled and unlabeled bacterial suspensions were fixed with 1% (vol/vol) formaldehyde in HEPES buffer pH 7.4 for 30 min at room temperature and analyzed using a BD Accuri C6 Plus flow cytometer (BD Biosciences, Sweden) in logarithmic mode. The forward scatter height (FSC-H) threshold was set at 25,000. Samples were diluted so as not to exceed an event rate of 3,500 events per second. Representative gating strategies and histograms are presented in Fig. S1. For bacterial suspensions, singlets were gated according to FSC-H and area. From singlets, the bacterial population was gated based on forward and side scatter area (SSC-A). At least 12,000 events were collected in the bacterial population gate for each sample. Unstained cells were used to define the background for each bacterial species. Histograms were then used to determine the median fluorescence intensity (MFI) of the whole population and the percentage of CellTrace CFSE-labeled cells within the population using BD Accuri C6Plus software.

### Confocal microscopic imaging of CellTrace-labeled biofilms

Visualization of biofilms was performed using a confocal Eclipse TE200 inverted microscope equipped for both spinning disk (CSDM) and laser scanning microscopy (CLSM), combined with NIS-Elements imaging software (version 5.02, Nikon Corp., Tokyo, Japan). For quantification, the surface coverage of the CellTrace CFSE stained area in SS biofilms was calculated as a percentage of the total area within a defined region of interest (ROI). Images were collected from three technical replicates of three independent experiments for each condition. For MS biofilms, an overlay of 4 color channels was used in CSDM to obtain both single-plane and multi-plane (Z-stack) images, with the Z-stack images having a slice depth of 0.1–0.3 µm. Percentage coverage by each species in the MS biofilms was determined using an identical ROI in all color channels of the single-plane images. The stained area for each species (corresponding to one color) was then calculated and presented as a percentage of the total stained area. Biofilms stained with BacLight LIVE/DEAD viability stain (Molecular Probes, Eugene, OR, USA) were imaged by CLSM using an argon laser (488 nm) for excitation and a long-pass 515/30 emission filter.

### Detachment studies of biofilm bacteria

For detachment studies, Gram-positive (*S. gordonii* and *A. odontolyticus*) and Gram-negative (*P. gingivalis* and *V. parvula*) bacteria were labeled with CellTrace CFSE and SS biofilms created as described above. After 4 hours (day 0), the starting inoculum in PRNM was replaced by flushing the flow-cell channel with an equal volume of PRNM supplemented with 20% equine serum (PRNM + serum) (Håtunalab, AB, Bro, Sweden). The medium was then replaced daily for the next 3 days by pipetting away the spent medium and replacing it with an equal volume of fresh medium (with no additional washing steps). Biofilms were imaged using CSDM on each day immediately after medium exchange. Cells in the starting inoculum, as well as the biofilm supernatants from days 1–3, were retained and fixed for FC. Experiments were carried out in triplicate using independent biological replicates with representative images shown.

### CellTrace staining and bacterial cell viability

To assess whether CellTrace staining was related to the cellular activity of the bacteria, SS biofilms of *S. gordonii* and *P. gingivalis* were prepared using diluted suspensions (1 µL loop in 2 mL PBS) as described above. Biofilms were maintained overnight in PRNM before being treated for 1 hour with either 70% (vol/vol) ethanol or 10% hydrogen peroxide. Control biofilms were maintained in PRNM. After treatment, biofilms were rinsed three times and stained *in situ* with either CellTrace CFSE for 1 hour or BacLight LIVE/DEAD viability stain for 5 min prior to imaging.

### Assessment of CellTrace dyes for multiplexing in MS biofilms

Assessment of CellTrace dyes as a tool for visualizing MS biofilms was carried out in two models related to oral disease: periodontitis (*P. gingivalis* [far red], *P. micra* [CFSE], *A. odontolyticus* [violet], and *S. gordonii* [yellow] and dental caries (*V. parvula* [far red], A. *naeslundii* [CFSE], L. *paracasei* [violet], and S. *mutans* [yellow]). Biofilms were prepared as described above. After attachment under stationary conditions for 4 hours, the PRNM was replaced with either fresh PRNM (caries model) or PRNM + serum (periodontitis model) with biofilms being maintained overnight before imaging with CSDM. Experiments were carried out in triplicate using independent biological replicates with representative images shown.

To assess the durability of the CellTrace dyes over time in MS biofilms, communities corresponding to the periodontitis model were created in PRNM with aliquots (200 µL) subsequently introduced into channels of IbiTreat VI slides. Slides were centrifuged briefly (1,000 rpm, 5 min) to facilitate attachment onto the slide surface for imaging purposes. After 4 hours, the PRNM was replaced with PRMN + serum and the medium was then changed daily for a further 3 days. Biofilms were imaged after 1 and 4 days. To examine changes in dye intensity over time, the input intensity range values used were identical for both days. To assess the bacterial viability after 4 days in the biofilm, parallel biofilms without CellTrace were maintained under similar conditions and visualized using Baclight LIVE/DEAD stain on day 4.

### Influence of environment on early biofilm formation

To assess the effect of different environmental conditions on bacterial attachment and early biofilm formation, MS communities corresponding to the periodontitis model were made in PBS, PRNM, or PRNM + serum. Biofilms were formed in IbiTreat VI slides as described previously and maintained for either 4 or 24 hours before imaging with CSDM using identical parameter settings.

### Statistical analysis

Statistical analysis was undertaken using GraphPad Prism 10 software.

## SUPPLEMENTAL MATERIAL

Figure S1 spectrum.00253-24-s0001.tiff Gating strategy.

Figure S2 spectrum.00253-24-s0002.tiff Histogram plots.

ASM does not own the copyrights to Supplemental Material that may be linked to, or accessed through, an article. The authors have granted ASM a non-exclusive, world-wide license to publish the Supplemental Material files. Please contact the corresponding author directly for reuse.
