# Supplementary figures and images for: A novel multiplex fluorescent-labeling method for the visualization of mixed-species biofilms in vitro
Source: Microbiol Spectr. 2024 May 24;12(7):e00253-24. doi: 10.1128/spectrum.00253-24 (PMC11218471; doi:10.1128/spectrum.00253-24)

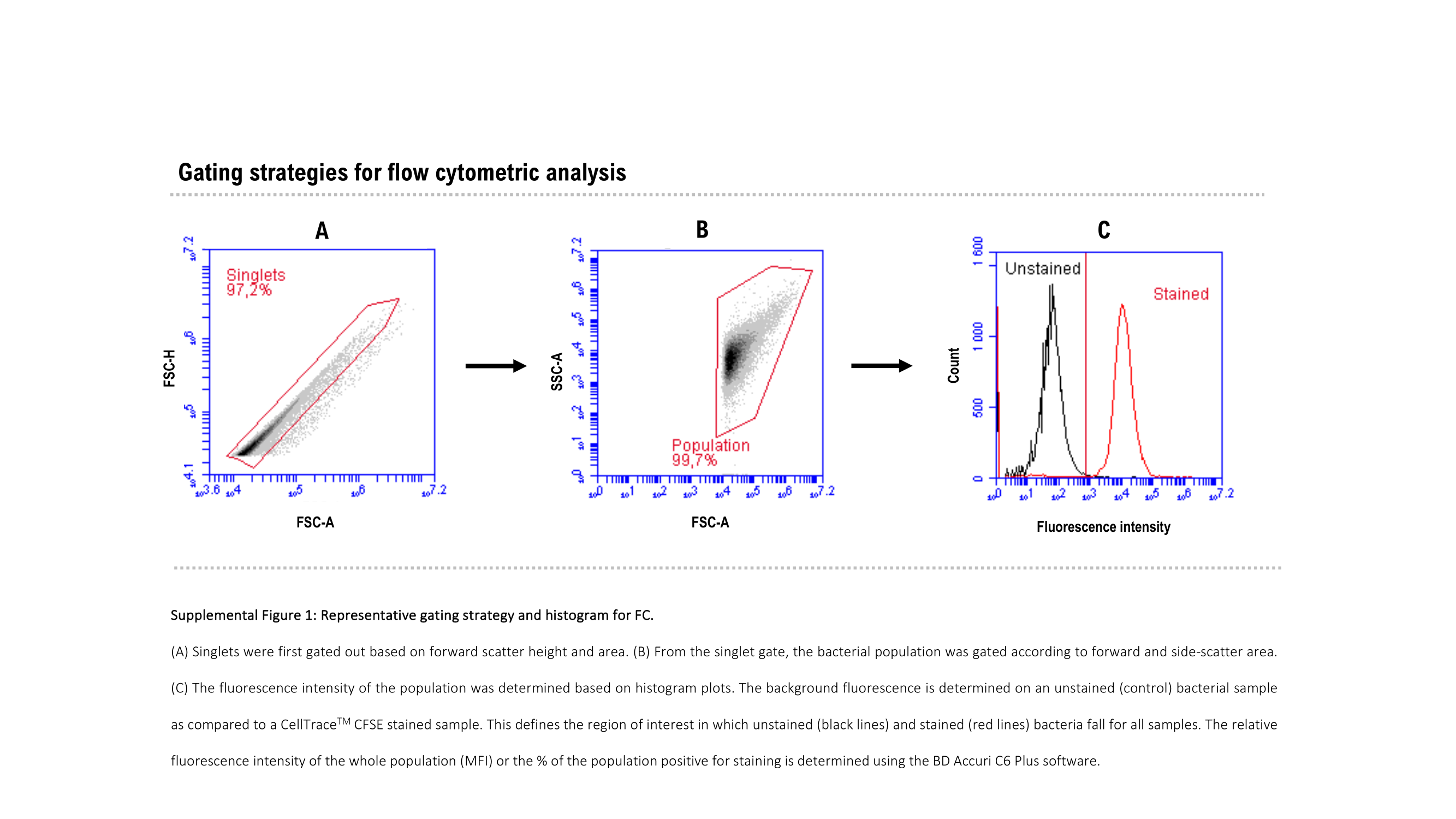

Supplement: Figure S1 — Gating strategy. [file spectrum.00253-24-s0001.tiff]

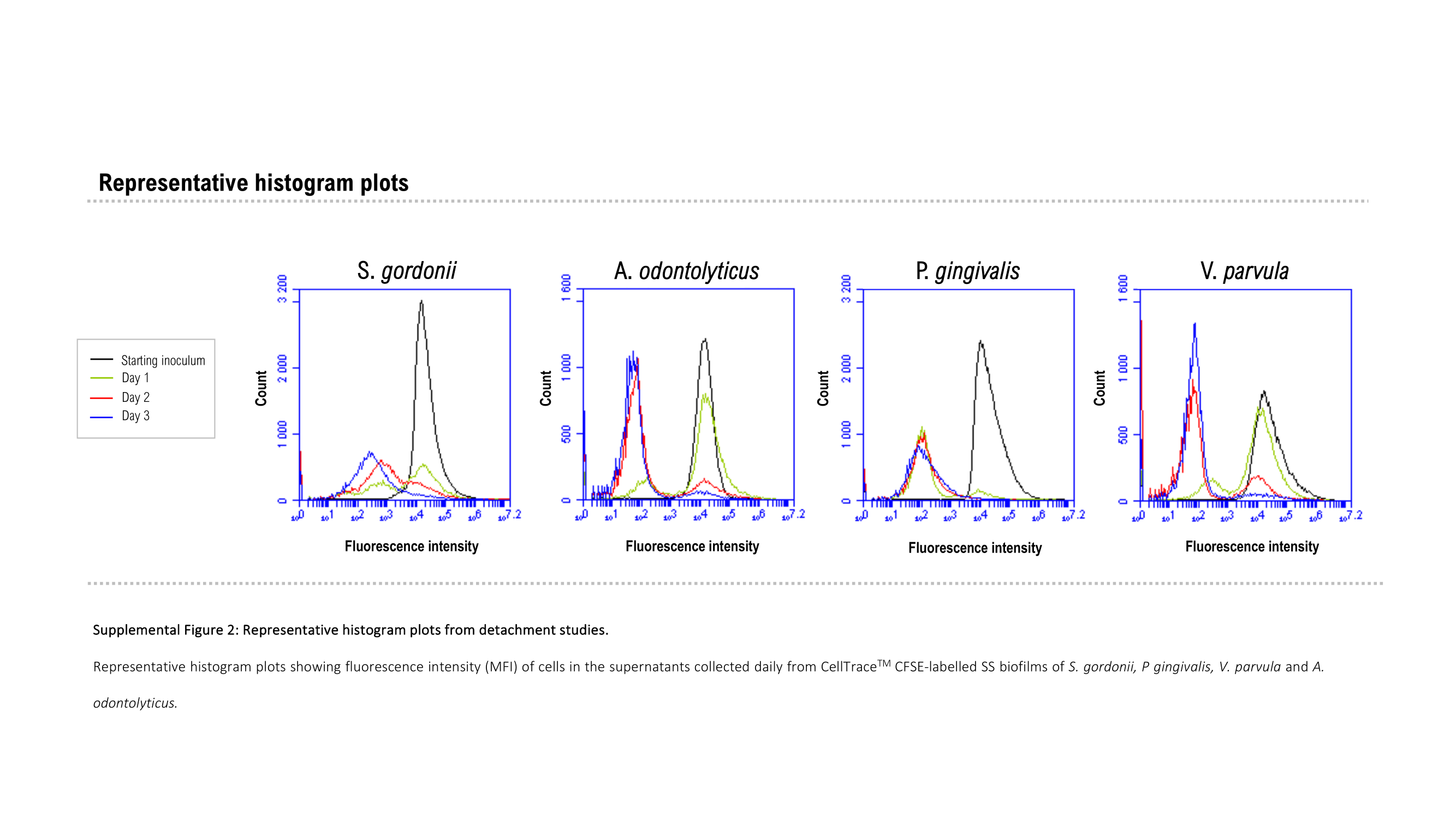

Supplement: Figure S2 — Histogram plots. [file spectrum.00253-24-s0002.tiff]
